# Correction: Modelling and prediction of the thermophysical properties of aqueous mixtures of choline geranate and geranic acid (CAGE) using SAFT-γ Mie

**DOI:** 10.1039/d0ra90058c

**Published:** 2020-05-22

**Authors:** Silvia Di Lecce, Georgia Lazarou, Siti H. Khalit, David Pugh, Claire S. Adjiman, George Jackson, Amparo Galindo, Lisa McQueen

**Affiliations:** Department of Chemical Engineering, Centre for Process Systems Engineering, Institute for Molecular Science and Engineering, South Kensington Campus, Imperial College London London SW7 2AZ UK a.galindo@imperial.ac.uk; Department of Chemistry, Molecular Sciences Research Hub, White City Campus, Imperial College London London W12 0BZ UK; Chemical Development, GSK 1250 S Collegeville Rd Collegeville PA 19426 USA

## Abstract

Correction for ‘Modelling and prediction of the thermophysical properties of aqueous mixtures of choline geranate and geranic acid (CAGE) using SAFT-γ Mie’ by Silvia Di Lecce *et al.*, *RSC Adv.*, 2019, **9**, 38017–38031. DOI: 10.1039/C9RA07057E

The authors regret the omission of one of the authors, David Pugh, from the original manuscript. The corrected list of authors and affiliations for this paper is as shown here.

In addition, we point readers to ref. 1 and 2, together with ref. 17–21 in the original paper, for a complete description of the association contribution to the SAFT-γ Mie equation of state.

The authors also wish to correct a number of typographical errors in Tables 3 and 4. The corrected Tables 3 and 4 are shown below; the letters and numbers in bold indicate the corrected values.

**Table:** Unlike dispersion interaction energies (*ε*_kl_/*k*_B_)/K and repulsive exponents *λ*^r^_kl_ for use within the SAFT-γ Mie group-contribution approach. CR indicates a combining rule is used to determine the value of the corresponding parameter. The unlike dispersion interactions indicated with CR are calculated using eqn (7) for uncharged groups and eqn (10) for charged groups. The combining rule is used to determine the value of *λ*^r^_kl_ is given in eqn (6). The unlike group diameters *σ*_kl_ are obtained using the combining rule given in eqn (5) in all cases

Group k	Group l	(*ε*_kl_/*k*_B_)/K	*λ* ^r^_kl_	Ref.	Group k	Group l	(*ε*_kl_/*k*_B_)/K	*λ* ^r^_kl_	Ref.
CH_3_/^adj^CH_3_	CH_3_/^adj^CH_3_	256.77	15.050	17	CH=	Cl^−^	CR	CR	This work
CH_3_/^adj^CH_3_	CH_2_/^adj^CH_2_	350.77	CR	17	COOH	COOH	405.78	8.0000	18
CH_3_/^adj^CH_3_	CH_2_=	333.48	CR	18	COOH	H_2_O	289.76	CR	19
CH_3_/^adj^CH_3_	CH=	252.41	CR	18	COOH	CH_2_OH	656.80	CR	19
CH_3_/^adj^CH_3_	COOH	255.99	CR	18	COOH	C=	609.87	CR	This work
CH_3_/^adj^CH_3_	H_2_O	358.18	100.00	19	COOH	COO^−^	405.78	8.0000	This work
CH_3_/^adj^CH_3_	CH_2_OH	333.20	CR	19	COOH	N^+^	CR	CR	This work
CH_3_/^adj^CH_3_	C=	281.40	CR	69	COOH	Na^+^	CR	CR	This work
CH_3_	COO^−^	255.99	CR	This work	COOH	K^+^	CR	CR	This work
^adj^CH_3_	COO^−^	509.37	CR	This work	COOH	Cl^−^	CR	CR	This work
CH_3_/^adj^CH_3_	N^+^	462.18	CR	This work	H_2_O	H_2_O	266.68	17.020	68
CH_3_/^adj^CH_3_	Na^+^	CR	CR	This work	H_2_O	CH_2_OH	353.37	CR	19
CH_3_/^adj^CH_3_	K^+^	CR	CR	This work	H_2_O	C=	310.91	8.0000	This work
CH_3_/^adj^CH_3_	Cl^−^	CR	CR	This work	H_2_O	COO^−^	171.61	CR	This work
CH_2_/^adj^CH_2_	CH_2_/^adj^CH_2_	473.39	19.871	17	H_2_O	N^+^	1481.3	21.217	This work
CH_2_/^adj^CH_2_	CH_2_=	386.80	CR	18	H_2_O	Na^+^	539.68	CR	20
CH_2_/^adj^CH_2_	CH=	459.40	CR	18	H_2_O	K^+^	376.25	CR	20
CH_2_/^adj^CH_2_	COOH	413.74	CR	18	H_2_O	Cl^−^	95.406	CR	20
CH_2_/^adj^CH_2_	H_2_O	423.63	100.00	19	CH_2_OH	CH_2_OH	407.22	22.699	19
CH_2_/^adj^CH_2_	CH_2_OH	423.17	CR	19	CH_2_OH	C=	799.66	CR	This work
CH_2_/^adj^CH_2_	C=	286.58	CR	69	CH_2_OH	COO^−^	656.80	CR	This work
CH_2_	COO^−^	413.74	CR	This work	CH_2_OH	N^+^	440.99	CR	This work
^**adj**^ **CH**_**2**_	COO^−^	780.24	CR	This work	CH_2_OH	Na^+^	CR	CR	This work
CH_2_/^adj^CH_2_	N^+^	348.30	CR	This work	CH_2_OH	K^+^	CR	CR	This work
CH_2_/^adj^CH_2_	Na^+^	CR	CR	This work	CH_2_OH	Cl^−^	CR	CR	This work
CH_2_/^adj^CH_2_	K^+^	CR	CR	This work	C=	C=	1500.0	8.0000	69
CH_2_/^adj^CH_2_	Cl^−^	CR	CR	This work	C=	COO^−^	609.87	CR	This work
CH_2_=	CH_2_=	300.90	20.271	18	C=	N^+^	CR	CR	This work
CH_2_=	CH=	275.75	CR	18	C=	Na^+^	CR	CR	This work
CH_2_=	COOH	**CR**	**CR**	This work	C=	K^+^	CR	CR	This work
CH_2_=	H_2_O	**387.25**	**94.463**	This work	C=	Cl^−^	CR	CR	This work
CH_2_=	CH_2_OH	**375.51**	CR	This work	COO^−^	COO^−^	**21.264**	8.0000	This work
CH_2_=	C=	**203.76**	CR	This work	COO^−^	N^+^	24.280	CR	This work
CH_2_=	COO^−^	CR	CR	This work	COO^−^	Na^+^	9.9125	CR	This work
CH_2_=	N^+^	CR	CR	This work	COO^−^	K^+^	23.999	CR	This work
CH_2_=	Na^+^	CR	CR	This work	COO^−^	Cl^−^	**47.154**	CR	This work
CH_2_=	K^+^	CR	CR	This work	N^+^	N^+^	**62.971**	**8.8971**	This work
CH_2_=	Cl^−^	CR	CR	This work	N^+^	Na^+^	CR	CR	This work
CH=	**CH=**	952.54	15.974	18	N^+^	K^+^	CR	CR	This work
CH=	COOH	453.13	CR	This work	N^+^	Cl^−^	61.989	CR	This work
CH=	H_2_O	332.21	17.309	This work	Na^+^	Na^+^	31.711	12.000	20
CH=	CH_2_OH	414.91	CR	This work	Na^+^	K^+^	CR	CR	This work
CH=	C=	1195.3	CR	69	Na^+^	Cl^−^	27.938	CR	20
CH=	COO^−^	453.13	CR	This work	K^+^	K^+^	90.097	12.000	20
CH=	N^+^	CR	CR	This work	K^+^	Cl^−^	61.010	CR	20
CH=	Na^+^	CR	CR	This work	Cl^−^	Cl^−^	113.77	12.000	20
CH=	K^+^	CR	CR	This work					

**Table:** Association energy *ε*^HB^_ab,kl_/*k*_B_ and bonding volume *K*^HB^_ab,kl_ parameters for use within the SAFT-γ Mie group-contribution approach. For groups with several site types, the interactions are symmetrical, *i.e.*, *ε*^HB^_ab,kl_ = *ε*^HB^_ba,lk_. Interactions not reported here are set to zero

Group k	Site *a* of group k	Group l	Site *b* of group l	(*ε*^HB^_ab,kl_/*k*_B_)/K	*K* ^HB^_ab,kl_/Å^3^	Ref.
COOH	H	COOH	H	6427.9	0.8062	18
COOH	e_1_	H_2_O	H	1451.8	280.89	19
COOH	e_2_	H_2_O	H	1252.6	150.98	19
COOH	H	H_2_O	e_1_	2567.7	270.09	19
COOH	e_1_	CH_2_OH	H	1015.5	21.827	19
COOH	e_2_	CH_2_OH	H	547.4**2**	53.150	19
COOH	H	CH_2_OH	e_1_	524.0**4**	14.017	19
H_2_O	e_1_	H_2_O	H	1985.4	101.69	68
H_2_O	e_1_	CH_2_OH	H	621.68	425.00	19
H_2_O	H	CH_2_OH	e_1_	2153.2	147.40	19
H_2_O	H	COO^−^	e_1_	802.2**1**	52.5**55**	This work
H_2_O	e_1_	N^+^	H	2783.7	15.536	This work
CH_2_OH	e_1_	CH_2_OH	H	2097.9	62.3**09**	19
CH_2_OH	e_1_	N^+^	H	1247.2	286.83	This work

The Royal Society of Chemistry apologises for these errors and any consequent inconvenience to authors and readers.

## Supplementary Material
